# Supplementary material for: HIVprotI: an integrated web based platform for prediction and design of HIV proteins inhibitors
Source: J Cheminform. 2018 Mar 9;10:12. doi: 10.1186/s13321-018-0266-y (PMC5845081; doi:10.1186/s13321-018-0266-y)
Supplement: Supplementary file 2 — Additional file 2. Source code of HIVProtI web server. [file 13321_2018_266_MOESM2_ESM.zip › HIVprotI_Source-code/hivprotI/weka-3-6-6/doc/weka/gui/ViewerDialog.html]

ViewerDialog


---


|  |  |  |
| --- | --- | --- |
| **Overview** \| **Package** \| **Class** \| **Tree** \| **Deprecated** \| **Index** \| **Help** \| **Weka's home** | |  |
| **PREV CLASS**   **NEXT CLASS** | **FRAMES**    **NO FRAMES**     **All Classes** |  |
| SUMMARY: NESTED \| FIELD \| CONSTR \| METHOD | DETAIL: FIELD \| CONSTR \| METHOD |  |


---


## weka.gui Class ViewerDialog

```
java.lang.Object
  java.awt.Component
      java.awt.Container
          java.awt.Window
              java.awt.Dialog
                  javax.swing.JDialog
                      weka.gui.ViewerDialog
```

**All Implemented Interfaces:**: java.awt.image.ImageObserver, java.awt.MenuContainer, java.io.Serializable, java.util.EventListener, javax.accessibility.Accessible, javax.swing.event.ChangeListener, javax.swing.RootPaneContainer, javax.swing.WindowConstants

---

``` public class ViewerDialog extends javax.swing.JDialog implements javax.swing.event.ChangeListener ```

A downsized version of the ArffViewer, displaying only one Instances-Object.

**Version:**
:   $Revision: 7059 $

**Author:**
:   FracPete (fracpete at waikato dot ac dot nz)

**See Also:**: `ArffViewer`, Serialized Form

---

**Nested Class Summary**

**Nested classes/interfaces inherited from class java.awt.Dialog**

`java.awt.Dialog.ModalExclusionType, java.awt.Dialog.ModalityType`

**Nested classes/interfaces inherited from class java.awt.Component**

`java.awt.Component.BaselineResizeBehavior`


| **Field Summary** | |
| --- | --- |
| `static int` | `APPROVE_OPTION`             Signifies an OK property selection |
| `static int` | `CANCEL_OPTION`             Signifies a cancelled property selection |

**Fields inherited from class java.awt.Dialog**

`DEFAULT_MODALITY_TYPE`

**Fields inherited from class java.awt.Component**

`BOTTOM_ALIGNMENT, CENTER_ALIGNMENT, LEFT_ALIGNMENT, RIGHT_ALIGNMENT, TOP_ALIGNMENT`

**Fields inherited from interface javax.swing.WindowConstants**

`DISPOSE_ON_CLOSE, DO_NOTHING_ON_CLOSE, EXIT_ON_CLOSE, HIDE_ON_CLOSE`

**Fields inherited from interface java.awt.image.ImageObserver**

`ABORT, ALLBITS, ERROR, FRAMEBITS, HEIGHT, PROPERTIES, SOMEBITS, WIDTH`


**Constructor Summary**

`ViewerDialog(java.awt.Frame parent)`
  
          initializes the dialog with the given parent


| **Method Summary** | |
| --- | --- |
| `Instances` | `getInstances()`             returns the currently displayed instances |
| `boolean` | `isChanged()`             returns whether the data has been changed |
| `void` | `setInstances(Instances inst)`             sets the instances to display |
| `int` | `showDialog()`             Pops up the modal dialog and waits for Cancel or OK. |
| `int` | `showDialog(Instances inst)`             Pops up the modal dialog and waits for Cancel or OK. |
| `void` | `stateChanged(javax.swing.event.ChangeEvent e)`             Invoked when the target of the listener has changed its state. |

**Methods inherited from class javax.swing.JDialog**

`getAccessibleContext, getContentPane, getDefaultCloseOperation, getGlassPane, getGraphics, getJMenuBar, getLayeredPane, getRootPane, getTransferHandler, isDefaultLookAndFeelDecorated, remove, repaint, setContentPane, setDefaultCloseOperation, setDefaultLookAndFeelDecorated, setGlassPane, setJMenuBar, setLayeredPane, setLayout, setTransferHandler, update`

**Methods inherited from class java.awt.Dialog**

`addNotify, getModalityType, getTitle, hide, isModal, isResizable, isUndecorated, setModal, setModalityType, setResizable, setTitle, setUndecorated, setVisible, show, toBack`

**Methods inherited from class java.awt.Window**

`addPropertyChangeListener, addPropertyChangeListener, addWindowFocusListener, addWindowListener, addWindowStateListener, applyResourceBundle, applyResourceBundle, createBufferStrategy, createBufferStrategy, dispose, getBufferStrategy, getFocusableWindowState, getFocusCycleRootAncestor, getFocusOwner, getFocusTraversalKeys, getGraphicsConfiguration, getIconImages, getInputContext, getListeners, getLocale, getModalExclusionType, getMostRecentFocusOwner, getOwnedWindows, getOwner, getOwnerlessWindows, getToolkit, getWarningString, getWindowFocusListeners, getWindowListeners, getWindows, getWindowStateListeners, isActive, isAlwaysOnTop, isAlwaysOnTopSupported, isFocusableWindow, isFocusCycleRoot, isFocused, isLocationByPlatform, isShowing, pack, paint, postEvent, removeNotify, removeWindowFocusListener, removeWindowListener, removeWindowStateListener, reshape, setAlwaysOnTop, setBounds, setBounds, setCursor, setFocusableWindowState, setFocusCycleRoot, setIconImage, setIconImages, setLocationByPlatform, setLocationRelativeTo, setMinimumSize, setModalExclusionType, setSize, setSize, toFront`

**Methods inherited from class java.awt.Container**

`add, add, add, add, add, addContainerListener, applyComponentOrientation, areFocusTraversalKeysSet, countComponents, deliverEvent, doLayout, findComponentAt, findComponentAt, getAlignmentX, getAlignmentY, getComponent, getComponentAt, getComponentAt, getComponentCount, getComponents, getComponentZOrder, getContainerListeners, getFocusTraversalPolicy, getInsets, getLayout, getMaximumSize, getMinimumSize, getMousePosition, getPreferredSize, insets, invalidate, isAncestorOf, isFocusCycleRoot, isFocusTraversalPolicyProvider, isFocusTraversalPolicySet, layout, list, list, locate, minimumSize, paintComponents, preferredSize, print, printComponents, remove, removeAll, removeContainerListener, setComponentZOrder, setFocusTraversalKeys, setFocusTraversalPolicy, setFocusTraversalPolicyProvider, setFont, transferFocusBackward, transferFocusDownCycle, validate`

**Methods inherited from class java.awt.Component**

`action, add, addComponentListener, addFocusListener, addHierarchyBoundsListener, addHierarchyListener, addInputMethodListener, addKeyListener, addMouseListener, addMouseMotionListener, addMouseWheelListener, bounds, checkImage, checkImage, contains, contains, createImage, createImage, createVolatileImage, createVolatileImage, disable, dispatchEvent, enable, enable, enableInputMethods, firePropertyChange, firePropertyChange, firePropertyChange, firePropertyChange, firePropertyChange, firePropertyChange, getBackground, getBaseline, getBaselineResizeBehavior, getBounds, getBounds, getColorModel, getComponentListeners, getComponentOrientation, getCursor, getDropTarget, getFocusListeners, getFocusTraversalKeysEnabled, getFont, getFontMetrics, getForeground, getHeight, getHierarchyBoundsListeners, getHierarchyListeners, getIgnoreRepaint, getInputMethodListeners, getInputMethodRequests, getKeyListeners, getLocation, getLocation, getLocationOnScreen, getMouseListeners, getMouseMotionListeners, getMousePosition, getMouseWheelListeners, getName, getParent, getPeer, getPropertyChangeListeners, getPropertyChangeListeners, getSize, getSize, getTreeLock, getWidth, getX, getY, gotFocus, handleEvent, hasFocus, imageUpdate, inside, isBackgroundSet, isCursorSet, isDisplayable, isDoubleBuffered, isEnabled, isFocusable, isFocusOwner, isFocusTraversable, isFontSet, isForegroundSet, isLightweight, isMaximumSizeSet, isMinimumSizeSet, isOpaque, isPreferredSizeSet, isValid, isVisible, keyDown, keyUp, list, list, list, location, lostFocus, mouseDown, mouseDrag, mouseEnter, mouseExit, mouseMove, mouseUp, move, nextFocus, paintAll, prepareImage, prepareImage, printAll, remove, removeComponentListener, removeFocusListener, removeHierarchyBoundsListener, removeHierarchyListener, removeInputMethodListener, removeKeyListener, removeMouseListener, removeMouseMotionListener, removeMouseWheelListener, removePropertyChangeListener, removePropertyChangeListener, repaint, repaint, repaint, requestFocus, requestFocusInWindow, resize, resize, setBackground, setComponentOrientation, setDropTarget, setEnabled, setFocusable, setFocusTraversalKeysEnabled, setForeground, setIgnoreRepaint, setLocale, setLocation, setLocation, setMaximumSize, setName, setPreferredSize, show, size, toString, transferFocus, transferFocusUpCycle`

**Methods inherited from class java.lang.Object**

`equals, getClass, hashCode, notify, notifyAll, wait, wait, wait`

**Field Detail**

### APPROVE\_OPTION

```
public static final int APPROVE_OPTION
```

:   Signifies an OK property selection

    **See Also:**: Constant Field Values

---


### CANCEL\_OPTION

```
public static final int CANCEL_OPTION
```

:   Signifies a cancelled property selection

    **See Also:**: Constant Field Values


**Constructor Detail**

### ViewerDialog

```
public ViewerDialog(java.awt.Frame parent)
```

:   initializes the dialog with the given parent

    **Parameters:**: `parent` - the parent for this dialog


**Method Detail**

### setInstances

```
public void setInstances(Instances inst)
```

:   sets the instances to display

---


### getInstances

```
public Instances getInstances()
```

:   returns the currently displayed instances

---


### isChanged

```
public boolean isChanged()
```

:   returns whether the data has been changed

    :   **Returns:**: true if the data has been changed

---


### stateChanged

```
public void stateChanged(javax.swing.event.ChangeEvent e)
```

:   Invoked when the target of the listener has changed its state.

    :   **Specified by:**: `stateChanged` in interface `javax.swing.event.ChangeListener`

---


### showDialog

```
public int showDialog()
```

:   Pops up the modal dialog and waits for Cancel or OK.

    :   **Returns:**: either APPROVE\_OPTION, or CANCEL\_OPTION

---


### showDialog

```
public int showDialog(Instances inst)
```

:   Pops up the modal dialog and waits for Cancel or OK.

    :   **Parameters:**: `inst` - the instances to display **Returns:**: either APPROVE\_OPTION, or CANCEL\_OPTION


---


|  |  |  |
| --- | --- | --- |
| **Overview** \| **Package** \| **Class** \| **Tree** \| **Deprecated** \| **Index** \| **Help** \| **Weka's home** | |  |
| **PREV CLASS**   **NEXT CLASS** | **FRAMES**    **NO FRAMES**     **All Classes** |  |
| SUMMARY: NESTED \| FIELD \| CONSTR \| METHOD | DETAIL: FIELD \| CONSTR \| METHOD |  |


---
